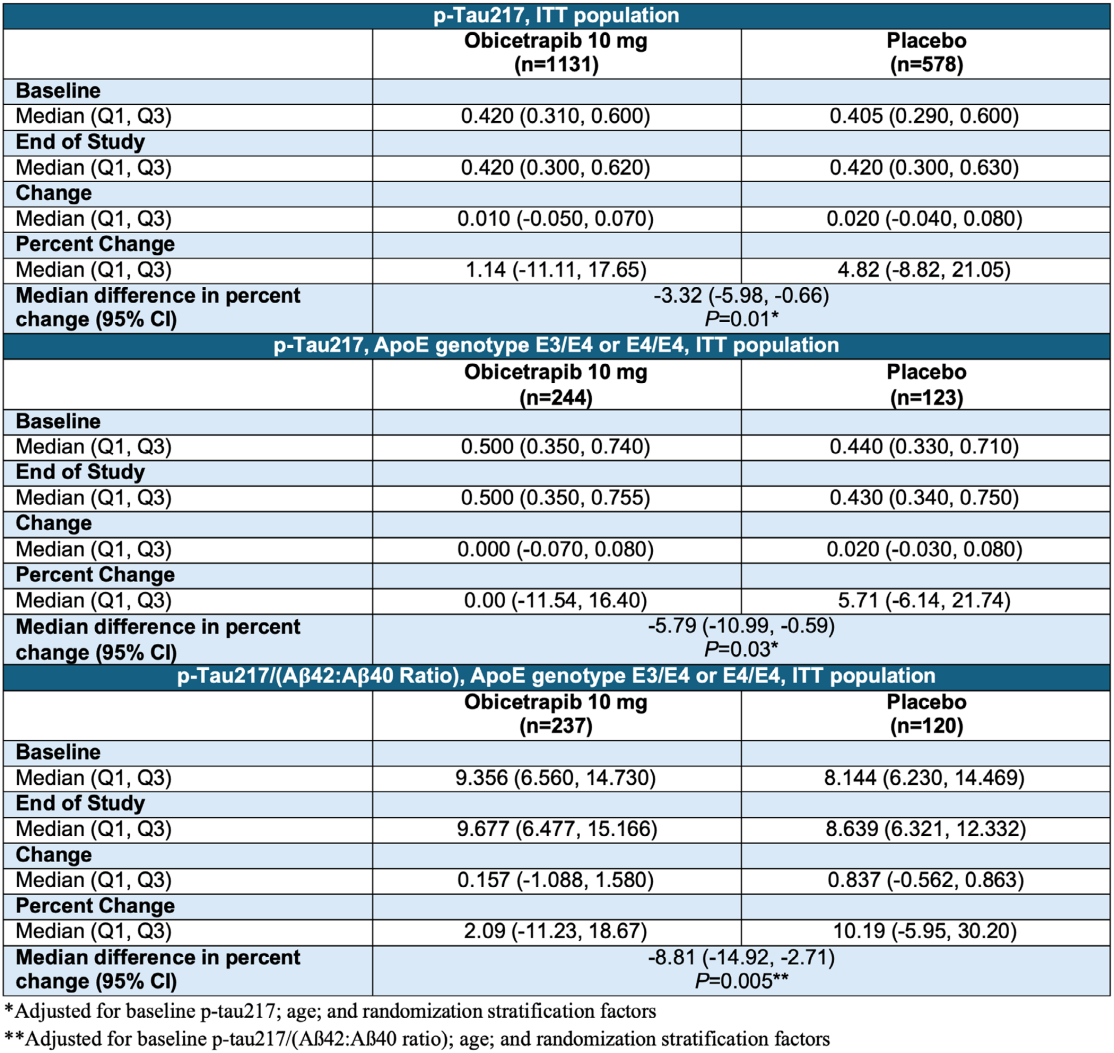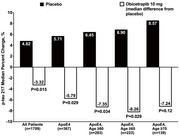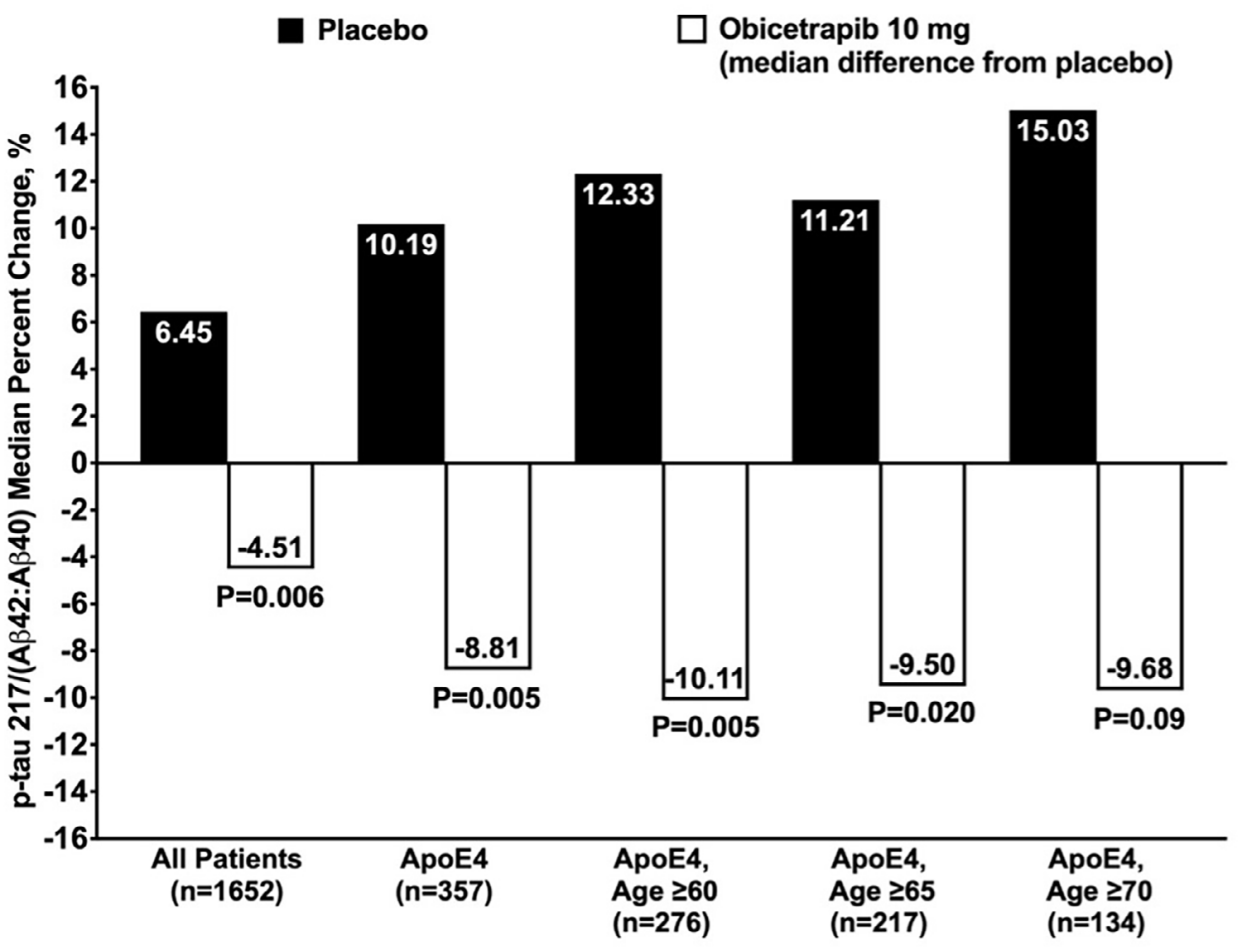# Effects of Obicetrapib, a Potent Oral CETP Inhibitor, on Alzheimer's Disease Biomarkers in 1727 Patients with cardiovascular disease

**DOI:** 10.1002/alz70861_108443

**Published:** 2025-12-23

**Authors:** Michael Davidson, Michael Szarek, Stephen Nicholls, Kosh K Ray, Douglas Kling, Marc Ditmarsch, Andrew Hsieh, Everard G.B. Vijverberg, Philip Scheltens, John Kastelein

**Affiliations:** ^1^ New Amsterdam Pharma, Naarden Netherlands; ^2^ University of Colorado, Anschutz, CO USA; ^3^ Victorian Heart Institute, Monash University, Melbourne Australia; ^4^ Imperial College, London UK; ^5^ Alzheimer Center Amsterdam, Neurology, Vrije Universiteit Amsterdam, Amsterdam UMC location VUmc, Amsterdam Netherlands

## Abstract

**Background:**

Cholesteryl ester transfer protein (CETP) regulates lipid metabolism, mediating the transfer of cholesteryl esters and triglycerides between LDL and HDL cholesterol particles. The CETP inhibitor, obicetrapib, reduces LDL‐C and increases HDL‐C. Genetic evidence and preclinical studies suggest that CETP inhibition might mitigate dementia and Alzheimer’s disease (AD) risk, particularly in individuals carrying the APOE4 allele. Dysfunctional cholesterol transport is a hallmark of AD, characterized by impaired lipidation of ApoE particles leading to neuroinflammation and neurodegeneration.

**Methods:**

BROADWAY study (NCT05142722), a Phase 3, double‐blind, placebo‐controlled trial evaluated obicetrapib 10 mg daily versus placebo over 12 months in individuals with established atherosclerosis cardiovascular disease. Participants (*n* =1727) were randomized 2:1 to treatment with obicetrapib or placebo. Plasma lipid levels including LDL‐C and HDL‐C were measured at baseline and at 12 months. AD plasma biomarkers, *p* ‐tau217, *p* ‐tau181, GFAP, NFL, and *p* ‐tau217/Aβ42:Aβ40 ratio, were also assessed at both time points. APOE status was determined.

**Results:**

The mean age of participants was 65 years, 33% were female. Obicetrapib significantly lowered LDL‐C by 33% and increased HDL‐C by 125% relative to placebo. Obicetrapib demonstrated significant attenuation of increase in the median plasma *p* ‐tau217 (1.1% obicetrapib vs. 4.8% with placebo; *p* =0.01) and *p* ‐tau217/Aβ42:Aβ40 ratio (2.7% obicetrapib vs. 6.5% placebo; *p* =0.006). At baseline, *p* ‐tau217 levels were higher in ApoE4 carriers compared with non‐carriers (0.47 pg/ml vs 0.39 pg/ml; (*p* <0.0001). ApoE4 carriers (*n* =367) showed greater benefit, with obicetrapib stabilizing *p* ‐tau217 levels (0% increase vs. 5.7% with placebo; *p* =0.03) and limiting increases in the *p* ‐tau217/Aβ42:Aβ40 ratio (2.1% vs. 10.2% placebo; *p* =0.005). Other AD biomarkers (*p* ‐tau181, GFAP, NFL) showed consistent favorable trends.

**Conclusions:**

This very large biomarker study showed that obicetrapib significantly slowed the progression of AD biomarkers over a period of 1 year, particularly *p* ‐tau217 and *p* ‐tau217/Aβ42:Aβ40 ratio, with most pronounced effects in older individuals carrying the APOE4 allele. These findings support the dual therapeutic potential of obicetrapib in addressing both cardiovascular and neurodegenerative disorders. Given its effects on the early evolution of β‐amyloid and tau pathology, obicetrapib represents a potential advancement in the prevention of AD, particularly in ApoE4 carriers who have an increased risks for both cardiovascular and AD pathologies.